# Classical and machine learning tools for identifying yellow-seeded *Brassica napus* by fusion of hyperspectral features

**DOI:** 10.3389/fgene.2024.1518205

**Published:** 2025-01-15

**Authors:** Fan Liu, Fang Wang, Zaiqi Zhang, Liang Cao, Jinran Wu, You-Gan Wang

**Affiliations:** ^1^ Hunan Provincial Key Laboratory of Dong Medicine, Hunan University of Medicine, Huaihua, China; ^2^ Key Laboratory of Intelligent Computing and Information Processing of Ministry of Education and Hunan Key Laboratory for Computation and Simulation in Science and Engineering, Xiangtan University, Xiangtan, China; ^3^ School of Mathematics and Physics, The University of Queensland, Brisbane, QLD, Australia

**Keywords:** rapeseed (Brassica napus), yellow-seeded, hyperspectral feature, logistic regression, partial least squares regression, machine learning

## Abstract

**Introduction:**

Due to its favorable traits-such as lower lignin content, higher oil concentration, and increased protein levels-the genetic improvement of yellow-seeded rapeseed has attracted more attention than other rapeseed color variations. Traditionally, yellow-seeded rapeseed has been identified visually, but the complex variability in the seed coat color of *Brassica napus* has made manual identification challenging and often inaccurate. Another method, using the RGB color system, is frequently employed but is sensitive to photographic conditions, including lighting and camera settings.

**Methods:**

We present four data-driven models to identify yellow-seeded *B. napus* using hyperspectral features combined with simple yet intelligent techniques. One model employs partial least squares regression (PLSR) to predict the R, G, and B color channels, effectively distinguishing yellow-seeded varieties from others according to globally accepted yellow-seed classification protocols. Another model uses logistic regression (Logit-R) to produce a probability-based assessment of yellow-seeded status. Additionally, we implement two intelligent models, random forest and support vector classifier to evaluate features selected through lasso-penalized logistic regression.

**Results and Discussion:**

Our findings indicate significant recognition accuracies of 96.55% and 98% for the PLSR and Logit-R models, respectively, aligning closely with the accuracy of previous methods. This approach represents a meaningful advancement in identifying yellow-seeded rapeseed, with high recognition accuracy demonstrating the practical applicability of these models.

## 1 Introduction

The quality of life and wellbeing of individuals are deeply influenced by the quality of rapeseed. The global adoption of cultivating “double high and double low” rapeseed-characterized by high oleic acid and oil content, along with low erucic acid and sulfur glycosides-underscores its vital significance.

During development, rapeseed contains chlorophyll, giving it a green hue. As the seeds mature, they exhibit colors ranging from black and reddish-brown to yellow. The seed coats of black and reddish-brown seeds accumulate pigments, while the transparent seed coats of yellow-seeded varieties reveal the embryo’s color. Studies indicate that yellow-seeded rapeseed has a shorter dormancy period, simpler germination, and higher oil content than black-seeded varieties, highlighting the breeding of yellow-seeded rapeseed as a valuable method to enhance oil content (Yang et al., 2021). Identifying yellow-seeded varieties of *Brassica juncea* and *Brassica campestris* is relatively straightforward, as the pure yellow phenotype is genetically stable (Li et al., 2012; Chen et al., 2015). However, stable, pure-yellow progeny have yet to be achieved in *Brassica napus* due to the complexity of seed coat color variations, including hetero-yellow patterns, such as yellow with black spots, patches, or brown rings. Additionally, the seed coat color of segregated progeny shows continuous variation (Liu, 1992; Auger et al., 2010; Qu et al., 2013). Consequently, accurately and efficiently determining the seed coat color of *B. napus* remains a critical and challenging task. Numerous studies address the identification of rapeseed color (Li et al., 2001; Somers et al., 2001; Zhang et al., 2006; Baetzel et al., 2003; Tańska et al., 2005; Li et al., 2012; Liu et al., 2005; Ye et al., 2018). For example, Li et al. (2001) estimated the yellow-seeded degree of *B. napus* through visual observation, a straightforward method but heavily reliant on the observer, leading to potentially inaccurate recognition. Somers et al. (2001) utilized light reflection to assess yellow-seeded color grade by measuring reflectance values and calculating the grain color index or light reflection values. While this method is more objective, it captures only single-dimensional color data, such as brightness, omitting rich information in the original material. To address this limitation, many scholars have focused on digital image analysis through the RGB color system (Zhang et al., 2006; Baetzel et al., 2003; Tańska et al., 2005; Li et al., 2012; Liu et al., 2005; Ye et al., 2018). However, the complex and similar coloration of the rapeseed epidermis makes precise color identification challenging, and the available techniques lack reliability and standardization. Accurate and efficient color measurement of yellow-seeded *B. napus* thus remains essential.

Recent advancements in chemometrics and computer technology have led to the development of near-infrared spectroscopy (NIRS), a technique combining both image and spectral data of the object. NIRS, known for its speed, non-destructive nature, and high efficiency, is widely used for the rapid, non-destructive analysis of agricultural products. Several studies have demonstrated its utility (Guo et al., 2019; Bu et al., 2023; Liang et al., 2023; Liu et al., 2021; Petisco et al., 2010; Sen et al., 2018; Liu et al., 2022; Zhang et al., 2020; Wei et al., 2020; Zhang et al., 2018; Jiang et al., 2017; Li et al., 2022; Jiang et al., 2018; He et al., 2022). For instance, Guo et al. (2019) used an NIRS imaging system (380–1,000 nm) to accurately quantify adulterated rice, while Bu et al. (2023) combined hyperspectral imaging with convolutional neural networks to create an intelligent model for sorghum variety recognition, surpassing existing models in accuracy. This technology has also been applied in rapeseed growth diagnostics. For example, Liu et al. (2021) developed a hyperspectral technique-based detection algorithm to predict the oleic acid content in *B. napus*. Petisco et al. (2010) examined visible and near-infrared spectra of *B. napus* and *B. juncea* with different oil levels, and Liu et al. (2022) developed an intelligent model that identified eleven *B. napus* varieties by integrating hyperspectral and spectral data, achieving a recognition accuracy of 93.71%. In another study, Wei et al. (2020) achieved 99.2% accuracy in classifying 15 soybean varieties through hyperspectral imaging, while Li et al. (2022) improved oleic acid content inversion in *B. napus* seeds by reducing hyperspectral redundancy.

As noted, identifying yellow-seeded *B. napus* is challenging due to the small seed size and complex seed coat color. Inconsistent RGB standards and labor-intensive calibration further complicate the task. The successful integration of hyperspectral technology in agriculture has paved the way for advancements in digital farming, inspiring us to apply it for yellow-seed identification. Furthermore, combining hyperspectral features with machine learning enhances remote sensing applications in smart agriculture, with hyperspectral analysis proving effective for modeling rapeseed aliphatic acid content. Hyperspectral sensing, a form of near-Earth remote sensing, provides a foundation for satellite- or drone-based agricultural monitoring. This technology facilitates tasks such as classification and inversion of land object parameters, which are challenging for traditional vision-based systems. Thus, our early-stage near-Earth remote sensing experiments are pivotal for advancing agricultural monitoring techniques.

In this study, we propose a novel approach to identifying yellow-seeded *B. napus* using hyperspectral imaging technology. We combine trilateral parameters derived from hyperspectral rapeseed imaging with spectral indices, applying partial least squares regression, logistic regression, random forest, and support vector classifiers. Importantly, the simplicity of spectral data collection addresses RGB calibration limitations. These intelligent models exhibit a commendable level of recognition accuracy. This study represents an initial exploration into rapeseed color classification through hyperspectral technology and machine learning, underscoring the significant potential of hyperspectral technology in tasks traditionally reliant on human vision systems.

## 2 Methods and materials

### 2.1 Materials

**FIGURE 1:**
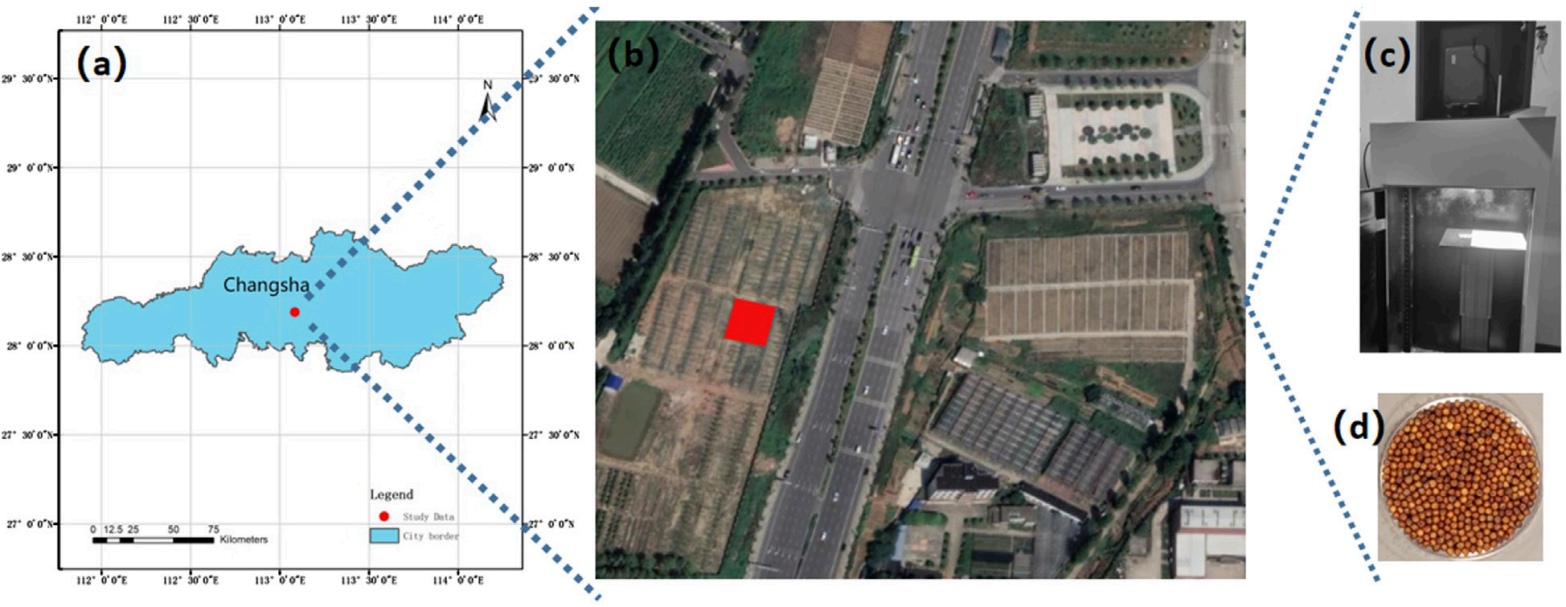
Location of the study area and hyperspectral collection environment. **(A)** Study area location in Changsha City, China; **(B)** Satellite map of Yunyuan Experimental Base (highlighted in red); **(C)** Hyperspectral data acquisition setup; **(D)** Region of Interest (ROI) for rapeseed sample.

For this study, we used two high oleic acid rapeseed varieties, namely, Xiangyou 708 and Xiangyou 710 (*B. napus. L*). These varieties were cultivated in the paddy fields of Yunyuan Experimental Base (28°23′N, 112°93′E, as illustrated in Figures 1A, B) located in Changsha City, Hunan Province, China. The region is situated in a subtropical monsoon climate, characterized by warm winters, hot summers, abundant rainfall primarily concentrated in the summer months, and distinct four seasons with relatively balanced seasonal distribution. In Changsha, the seasonal pattern features longer summers and winters, while spring and autumn are relatively shorter. And the average annual temperature is 17.2°C, the accumulated annual temperature is approximately 5,457°C, and the average yearly precipitation is 1361.6 mm. The crop rotation scheme involved black loam soil with rice as the preceding crop. Planting occurred in September 2020, with harvesting in April 2021.

**Remark 1:** This study represents a preliminary exploration into color classification of rapeseed seeds through integrating hyperspectral technology and machine learning methods. Recognizing the attributes of yellow-seeded varieties-such as high oil content, elevated polyphenol levels, enhanced nutritional value, and precise harvest time determination-we aim to employ the proposed model for identifying yellow-seeded rapeseed. Accordingly, we focus on the established varieties Xiangyou 708 and Xiangyou 710, which have been widely cultivated and promoted in Hunan Province, China.

### 2.2 Data collection

We utilized the SOC710 portable hyperspectral imager (wavelength range: 380–1,091 nm; resolution: 4.9458 nm) along with its darkroom system, manufactured by Surface Optics Corporation, United States, to collect spectral data. The optical obscura, as shown in Figure 1C, was maintained in dark conditions. Measuring 50×60×120 ${\text{cm}}^{3}$ , the obscura featured a movable base, a cooling device, and diffusely reflective coating inside. Equipped with a large and small task silo, a lifting table, and four groups of 70W halogen light sources adjustable in position, the spectrometer was mounted at the top small silo with a vertical lens at a distance of 370 mm from the rapeseed samples. A total of 300 rapeseed samples were selected (see Figure 1D), and each seed’s spectral reflectance was measured five times randomly, with the average taken as the sample reflectance. Additionally, the RGB color information of each sample was recorded.

### 2.3 Methods and indicators

Feature selection and modeling method are the key components of our seed identification model. First, the spectral index is commonly used as a reliable indicator in crop remote sensing for seed quality assessment. It enhances crop spectral information while effectively reducing noise and environmental interference by combining two or more spectral reflectances. Spectral indices provide rich data on rapeseed (Zhang et al., 2017; Hong et al., 2019; Bai et al., 2022). In this study, we employ three indices-ratio spectral index (RSI), difference spectral index (DSI), and normalized spectral index (NDSI), calculated for wavelengths 400–1,000 nm:

$${\text{RSI}}_{\left(\lambda_{1},\lambda_{2}\right)}=\frac{R_{\lambda_{1}}}{R_{\lambda_{2}}},$$ (1)

$${\text{DSI}}_{\left(\lambda_{1},\lambda_{2}\right)}=R_{\lambda_{1}}-R_{\lambda_{2}},$$ (2)

and

$${\text{NDSI}}_{\left(\lambda_{1},\lambda_{2}\right)}=\frac{R_{\lambda_{1}}-R_{\lambda_{2}}}{R_{\lambda_{1}}+R_{\lambda_{2}}}.$$ (3)

We also consider trilateral parameters-covering blue (490–530 nm), yellow (560–640 nm), and red (680–760 nm) edges-as additional hyperspectral features containing vital rapeseed information. Our study includes 23 representative parameters (see Table 1) encompassing characteristics such as location, amplitude, area, normalization, ratios, and numerical attributes (Peng et al., 2020; Sibanda et al., 2019).

**TABLE 1:** Hyperspectral trilateral characteristic parameter calculation formulas.

Name	Description or calculation
Db	Spectral maximum value of first derivative in blue edge (490–530 nm)
BDb	Bands corresponding to maximum first derivative value in blue edge
SDb	Square of blue edge: integration of first derivative over blue edge range
Dbmin	Minimum first derivative value in blue edge
NDb	Normalized blue-edge index, (Db-Dbmin)/(Db + Dbmin)
Dy	Spectral maximum value of first derivative in yellow edge (560–640 nm)
BDy	Bands corresponding to maximum first derivative value in yellow edge
SDy	Square of yellow edge: integration of first derivative over yellow edge range
Dymin	Minimum first derivative value in yellow edge
NDy	Normalized yellow-edge index, (Dy-Dymin)/(Dy + Dymin)
Dr	Spectral maximum value of first derivative in red edge (680–760 nm)
BDr	Bands corresponding to maximum first derivative value in red edge
SDr	Square of red edge: integration of first derivative over red edge range
Drmin	Minimum first derivative value in red edge
NDr	Normalized red-edge index, (Dr-Drmin)/(Dr + Drmin)
Srb	SDr/SDb
Sry	SDr/SDy
Syb	SDy/SDb
NBDb	(SDr-SDb)/(SDr + SDb)
Nry	(SDr-SDy)/(SDr + SDy)
Nby	(SDb-SDy)/(SDb + SDy)
Kur	Kurtosis of red-edge first derivative curve
Ske	Skewness of red-edge first derivative curve

These spectral indicators provide crucial information for characterizing seed color and are thus used in feature combinations for modeling.

To enable effective yellow-seeded rapeseed identification, we employ two well-established mathematical models. First, partial least squares regression (PLSR) (Meacham-Hensold et al., 2019) combines advantages of multiple linear regression, principal component analysis, and conventional correlation analysis and is commonly applied in spectral research. PLSR creates a linear regression model by projecting independent and response variables into a new space. Here, we use PLSR to predict the values of the three color channels [red (R), green (G), and blue (B)] for differentiating yellow-seeded from non-yellow-seeded rapeseeds.

Additionally, since generalized linear regression is suited for dichotomous classification, we apply logistic regression (Logit-R) to distinguish yellow from non-yellow-seeded rapeseed. The Logit-R model, a widely used machine learning technique, derives prediction parameters from training data and uses these to calculate a data segmentation hyperplane for classification (Broeckx et al., 2018; Lin et al., 2014; Munshi et al., 2014).

## 3 Results and discussion

### 3.1 Model preparation

#### 3.1.1 Labeling of yellow-seeded rapeseed samples

To classify the yellow and non-yellow rapeseed samples, we first labeled the 300 seed samples as yellow or non-yellow. This classification was based on internationally recognized guidelines using R, G, and B color channel thresholds (Zhang et al., 2006; Baetzel et al., 2003; Tańska et al., 2005), defined as follows:

$$Y_{R}=\left\{\begin{array}{cc} 0\; & R\notin S_{R}\\ 1\; & R\in \left\{S_{R}|164.1<R<245.3\right\}, \end{array}\right.$$ (4)

$$Y_{G}=\left\{\begin{array}{cc} 0\; & G\notin S_{G}\\ 1\; & G\in \left\{S_{G}|101.1<G<226.2\right\}, \end{array}\right.$$ (5)

$$Y_{B}=\left\{\begin{array}{cc} 0\; & B\notin S_{B}\\ 1\; & B\in \left\{S_{B}|9.1<B<92.2\right\}, \end{array}\right.$$ (6)

and

$$Y=Y_{R}\cap Y_{G}\cap Y_{B}=\left\{\begin{array}{c} 0,\text{otherwise}\;\\ 1,R\in S_{R},G\in S_{G},B\in S_{B}.\; \end{array}\right.$$ (7)

Here, Y=1 signifies yellow-seeded, while Y=0 denotes non-yellow-seeded. This labeling process resulted in 103 yellow-seeded and 197 non-yellow-seeded samples. Table 2 summarizes the RGB statistics for the 300 samples.

**TABLE 2:** Statistical characteristics of R/G/B values.

	Number of Samples	Red value		Green value		Blue value	
		Mean	Std.	Mean	Std.	Mean	Std.
Yellow	103	202.786	16.737	135.796	17.443	63.747	6.065
Non-yellow	197	118.426	31.735	79.061	9.63	63.035	6.673

#### 3.1.2 Spectral index feature extraction

**FIGURE 2:**
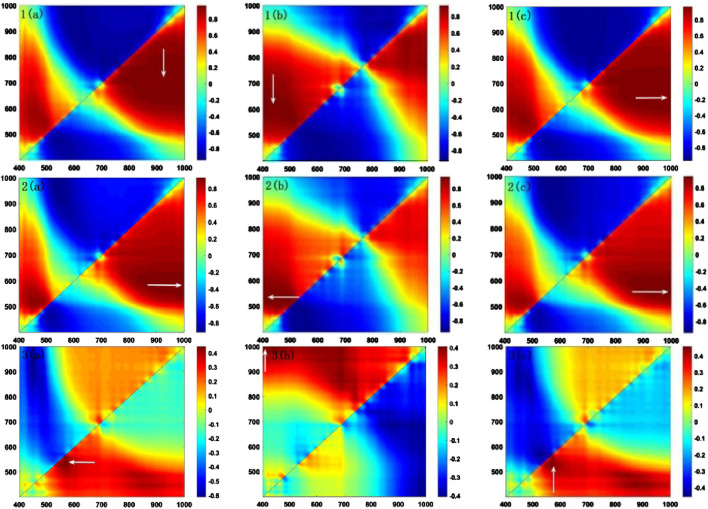
Correlation analysis between spectral indices and RGB values. Each heatmap highlights the highest correlation coefficient with an arrow. The top panels (label 1), middle panels (label 2) and bottom panels (label 3) denote R-value; 2 G-value; 3 B-value, respectively. **(A)** RSI; **(B)** DSI; **(C)** NDSI.

The spectral indices in Equations 1–3 capture various aspects of hyperspectral information. Selecting optimal band combinations is critical to obtaining the three spectral indices that best represent the red, green, and blue color information. A correlation analysis was conducted between the spectral indices and RGB values, with half of the samples used to distinguish between training and test sets, with 100 independent repetitions to minimize random noise effects. The highest average correlation coefficients for each spectral index, along with corresponding band combinations, are shown in Table 3. Figure 2 illustrates one of the implementations.

**TABLE 3:** Most correlated bands for spectral index.

Spectral Index	Red value		Green value		Blue value	
	$\left(\lambda_{1},\lambda_{2}\right)$	CC	$\left(\lambda_{1},\lambda_{2}\right)$	CC	$\left(\lambda_{1},\lambda_{2}\right)$	CC
RSI	(705, 910)	$0.9673^{**}$	(582, 983)	$0.9278^{**}$	(534, 571)	$0.4449^{**}$
DSI	(418, 610)	$0.9165^{**}$	(421, 536)	- $0.9440^{**}$	(419, 994)	- $0.4068^{**}$
NDSI	(646, 995)	$0.9660^{**}$	(568, 988)	$0.9380^{**}$	(531, 571)	$0.4563^{**}$

**significance at 0.01 level.

#### 3.1.3 Trilateral parameter feature extraction

**FIGURE 3:**
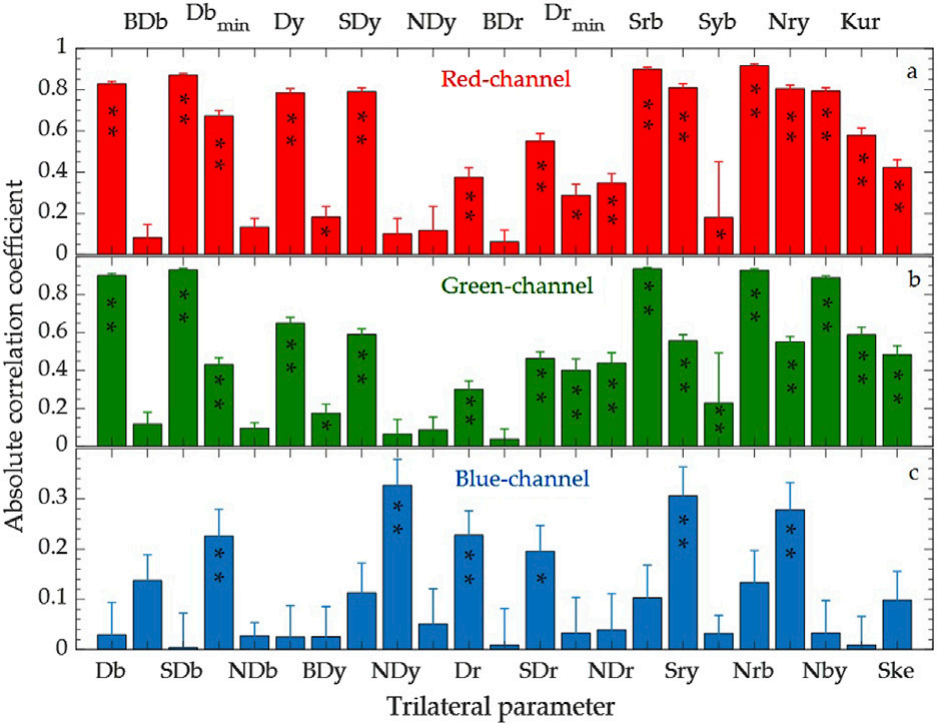
Absolute correlation coefficients between trilateral parameters and color channels. **(A)** Red channel, **(B)** Green channel, **(C)** Blue channel. Error bars represent standard deviations from 100 repetitions. * and ** denote significance at the 0.05 and 0.01 levels, respectively.

We also performed a correlation analysis between trilateral parameters and RGB color channels. A random selection of 150 samples with 100 repetitions was used. Figure 3 displays the absolute average correlation coefficients of the 23 trilateral parameters with red, green, and blue channels. For the red channel, the top three parameters are Nrb, Srb, and SDb, which are identical to those for the green channel. For the blue channel, the top three are Sry, Nry, and Dr, with lower correlation coefficients than the red and green channels but significant at the 0.01 level.

Based on the above correlation analysis, we identified optimal feature parameters for each channel. We subsequently combined the three spectral indices with the top three trilateral parameters to create feature sets for predicting the RGB values of test samples.

### 3.2 Modeling of rapeseed yellow-seeded identification

In this subsection, we establish four yellow-seed identification models by using PLSR, Logistic regression (Logit-R), and two machine learning methods, i.e., support vector classifier (SVC) and random forest. In each model, the 300 samples are randomly divided into two groups, namely, M training samples and (300−M) test samples, where M varies from 150 to 240 with step size 10. We train the models by the samples in the training set and test the model on the test set. In practice, we first input the feature combination of three spectral indexes and top-three trilateral parameters of the M training samples and the corresponding labeled yellow or non-yellow seeded (values of R/G/B or *Y*) to train the modes. Then, the feature combination of the 300−M test samples is input into the trained model to predict the result. In the end, the recognition accuracy (RA) is calculated according to the predicted results and the labeled results. To eliminate the interference of random noise, the above process is repeated 100 times to acquire the average RA.

#### 3.2.1 Predication modeling based on PLSR

According to the corn idea of PLSR, the arguments can be input into the model directly regardless of whether multi-collinearity or not. Therefore, we use the six parameters (the three spectral indexes and the top three trilateral parameters) of training samples and the corresponding R/G/B values to train the PLSR model first (constructing regression model to estimate the values of red, green, and blue, denoted by $\hat{R},\hat{G}$ , and $\hat{B}$ , respectively). Taking the M=150 (it means there are randomly chosen 150 training samples and 150 test samples) as an example, the regression estimation is,

$$\left\{\begin{array}{cc} \hat{R}=\; & 192.76+6.64\text{SDb}-29.05\text{Srb}+10.63\text{Nrb}+75.79{\text{RSI}}_{\left(705,910\right)}\\ \; & +126.53{\text{DSI}}_{\left(418,610\right)}-130.96{\text{NDSI}}_{\left(646,995\right)},\\ \hat{G}=\; & 171.39+33.79\text{SDb}+13.69\text{Srb}+23.13\text{Nrb}-90.19{\text{RSI}}_{\left(583,983\right)}\\ \; & +104.84{\text{DSI}}_{\left(421,536\right)}+148.18{\text{NDSI}}_{\left(568,988\right)},\\ \hat{B}=\; & 45.53-1.69\text{SDb}+0.41\text{Srb}+0.09\text{Nrb}+4.65{\text{RSI}}_{\left(534,571\right)}\\ \; & -11.79{\text{DSI}}_{\left(419,994\right)}+7.04{\text{NDSI}}_{\left(531,571\right)}.\; \end{array}\right.$$ (8)

**Remark 2.** Here, we approached false positives and false negatives with equal consideration. It is indeed accurate to acknowledge that lower error rates can also bring significant losses due to distinct economic implications associated with false negatives and false positives. Moving forward, we plan to enhance our color identification model by incorporating the economic impact of oil content.

**FIGURE 4:**
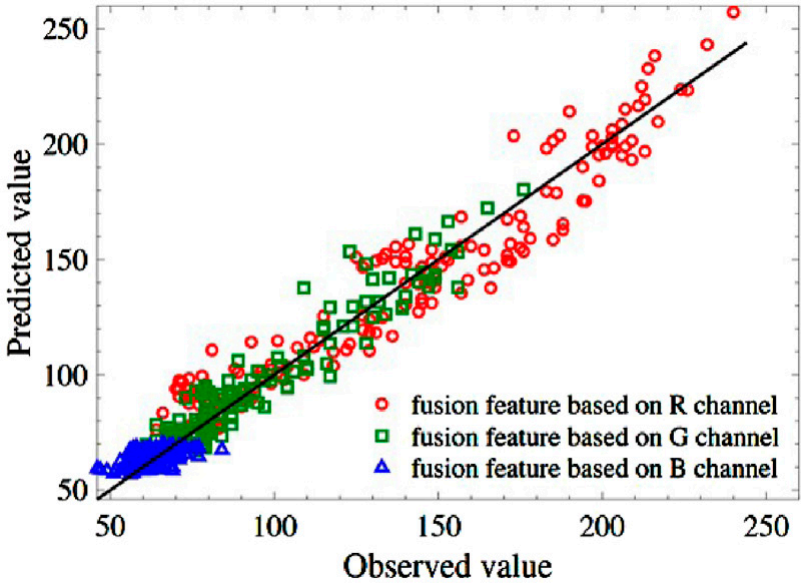
Comparison of predicted value and observed value of R, G, and B. The black solid line denotes the theoretical $\hat{Y}=Y$ .

Next, the same arguments of the 150 test samples are input into Equation 8 to inverse the R, G, and B values respectively. The predicted values and the labeled results are shown in Figure 4. The blue values locate a relatively narrow range compared to the red and green values, but they are all around the theoretical line of $\hat{Y}=Y$ .

Then, using the predicted values of R/G/B, the test samples can be classified into yellow-seeded and non-yellow-seeded according to Equations 4–7. Table 4 shows the confusion matrix for two different numbers of training samples, M=150 (150 test samples) and M=240 (60 test samples), respectively. The small number of misclassified samples is satisfactory.

**TABLE 4:** The identification results on the test set based on PLSR.

150 samples	Yellow	Non-yellow	60 samples	Yellow	Non-yellow
Yellow	45	6	Yellow	21	1
Non-yellow	4	95	Non-yellow	1	37

**FIGURE 5:**
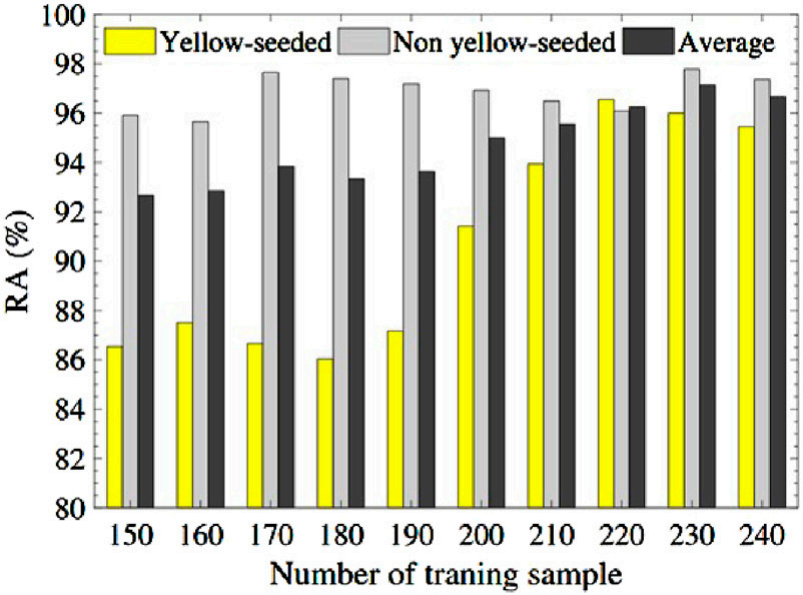
Recognition accuracy based on the PLSR model.

Varying M from 150 to 240, the RA of yellow-seeded, non-yellow-seeded, and mixed are calculated on the test set. As seen from Figure 5, the RA of the non-yellow-seeded is higher than that of the yellow-seeded. Naturally, the average RA tends to increase with the increase of training samples, however, it is located in the narrow interval of (92.32%, and 96.55%), which confirms the PLSR-based model with fusion feature of spectral index and trilateral parameters is workable.

#### 3.2.2 Recognition modeling based on Logit-R

**FIGURE 6:**
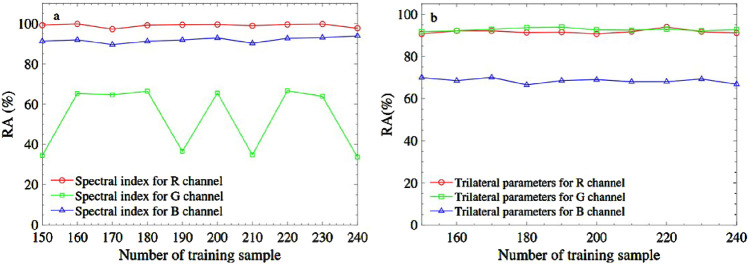
The recognition accuracy brought from R, G, and B channels based on different fusion features. **(A)** is spectral index combination and **(B)** is trilateral parameters combination.

Similar to the PLSR model, we first use the fusion feature and the corresponding labeled seed attribute of training samples to train the Logit-R model. Then, the fusion feature of the test samples is input into the trained model to predict the discriminant probability and further calculate the classification accuracy. What’s different from the PLSR model is the label of seed attribute is a Boolean variable of Y=1 (denotes yellow-seeded) and Y=0 (denotes non-yellow-seeded). To compare the model performance brought by the spectral index and trilateral parameters, the two kinds of features are input into the model respectively. To do so, the three spectral indexes and top-three trilateral parameters related to the highest correlation coefficient for the R/G/B channels are used as feature combinations, respectively to calculate the RA, as shown in Figure 6. In the Logit-R model, the regular coefficient is set at 0.2, the maximum iterations are set at 300, the learning rate is set at 0.01, and the threshold is set at 0.5. As seen in Figure 6, the RA obtained from the spectral index fusion for the green channel and the trilateral parameters for the blue channel is comparatively low. However, using the fusion feature of the three spectral indexes based on the red channel can reach as high as 98% RA, which is higher than other fusion features significantly, even higher than the result from the six-feature fusion based on the PLSR model. In addition, the high RA is regardless of the number of training samples. In this regard, the three-spectral index fusion for the red channel based on Logit-R is suggested to identify the rapeseed yellow seeded.

**FIGURE 7:**
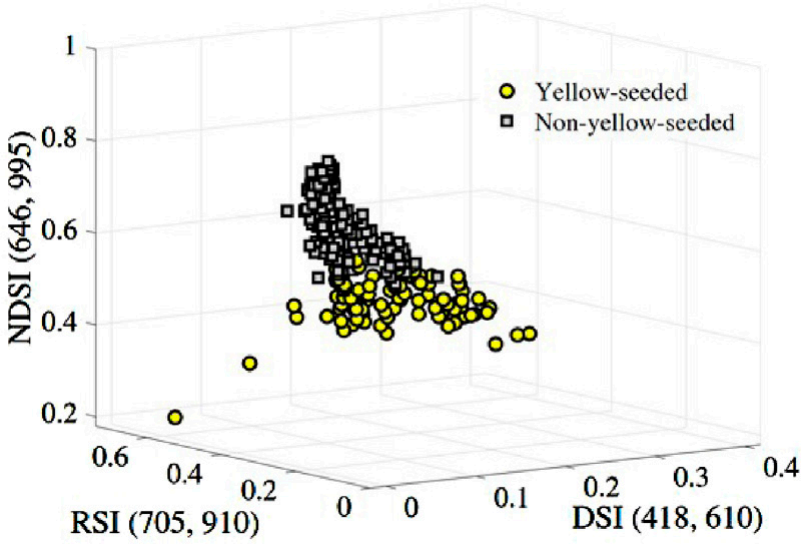
Two-classification of the yellow and non-yellow-seeded samples based on the feature combination {RSI(705,910),DSI(418,610),NDSI(646,995)} .

Following, let’s show how the Logit-R model using three-spectral indexes combination {RSI(705,910),DSI(418,610),NDSI(646,995)} for classifying the yellow and non-yellow seeded. Taking M=150 as an example. Before that, we placed the randomly selected 150 test samples in the three-dimensional space constructed by the above spectral indexes, as shown in Figure 7. The yellow-seeded and non-yellow-seeded are clustered together visually, respectively and separated from each other.

Now, we hope to find a segmentation hyperplane to separate the above two types of samples. To do so, firstly, we input the remaining 150 samples including the spectral index combination and the corresponding labeled Y = 0/1 to train the Logit-R model. The trained discriminant function is

$$h=\frac{1}{1+\exp\left(-3.3589\text{RSI}+4.3883\text{DSI}-5.1165\text{NDSI}\right)}.$$

**FIGURE 8:**
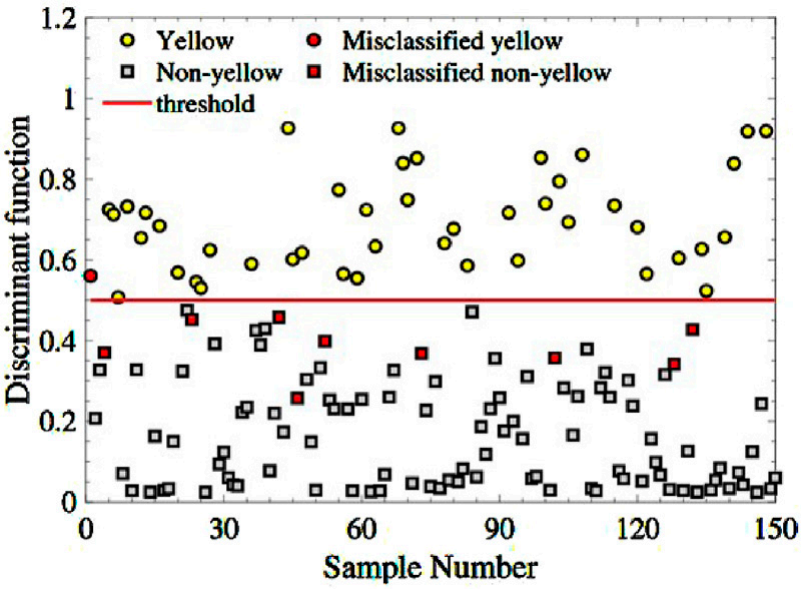
The classification of the yellow and non-yellow-seeded samples based on the feature combination {RSI(705,910),DSI(418,610),NDSI(646,995)} .

Then, inputting the three-spectral indexes combination of the 150 remaining samples into the trained model. Figure 7 depicts the sample distribution of four categories: yellow-seeded (yellow dots), non-yellow-seeded (grey squares), misclassified yellow-seeded samples (red dots), and misclassified non-yellow-seeded (red squares). As seen in Figure 8, there are only one yellow sample is misclassified and 9 non-yellow samples are misclassified. The RA of yellow-seeded and non-yellow-seeded is 97.78% and 91.43%, respectively, and the average RA is 93.33%.

#### 3.2.3 Predication results with machine learning methods

In this section, we employ two machine learning techniques: random forest Breiman (2001) and support vector classifier (SVC) Pisner and Schnyer (2020) to perform a classification task aimed at identifying the yellow seed. Differing from the preceding feature selection methods, we enhance our feature pool by integrating the original hyperspectral reflectances, consisting of nine spectral indexes calculated according to Table 3, and 23 trilateral parameters. This augmentation results in a total of 633 features.

To extract pivotal attributes and eliminate redundant data, we employ lasso-penalized logistic regression with the R package ‘glmnet’ (Friedman et al., 2010). The parameter λ , which balances the penalty and loss terms, is fine-tuned to maximize the “auc” index. Upon tuning, λ is set at 0.0062. Consequently, we identified 10 key features out of the initial 633. These features include the original hyperspectral values at wavelengths 394, 415, 416, 417, and 418, ${\text{NDSI}}_{\left(568,988\right)}$ (a spectral index), as well as Yep, Dymin, SDr, and ske (trilateral parameters).

According to these selected ten features, we conduct random forest and support vector classifier algorithms via R packages ‘rpart’ (Therneau et al., 2015) and ‘e1071’ (Meyer et al., 2024), respectively, by default settings with different training sizes (M: 150–240). The prediction results in test sets for the recognition task are reported in Table 5.

**TABLE 5:** Results of recognition accuracies with machine learning methods.

Training size M	Random forest			Support vector classifier		
	Average	Non-yellow	Yellow	Average	Non-yellow	Yellow
150	96.52%	98.31%	93.25%	97.99%	99.25%	95.68%
160	96.56%	98.23%	93.52%	97.87%	99.18%	95.50%
170	96.65%	98.42%	93.49%	97.98%	99.26%	95.70%
180	96.60%	98.34%	93.49%	98.07%	99.32%	95.81%
190	96.63%	98.38%	93.53%	97.98%	99.20%	95.83%
200	96.70%	98.55%	93.51%	98.00%	99.14%	96.03%
210	96.77%	98.42%	93.91%	97.99%	99.02%	96.21%
220	96.70%	98.35%	93.90%	97.94%	98.94%	96.23%
230	96.75%	98.34%	94.15%	98.03%	99.07%	96.33%
240	96.92%	98.57%	94.26%	98.08%	99.00%	96.61%

In comparing the recognition accuracies achieved by random forest and SVC methods, several key observations emerge. Regarding average accuracy, random forest demonstrates results ranging from approximately 96.52%–96.92%, while SVC exhibits a slightly higher range of around 97.87%–98.08%. Both methods exhibit an increase in accuracy with larger training sizes, with random forest’s accuracy gradually ascending and SVC’s accuracy following a similar trend, albeit with minor fluctuations.

In terms of category-specific accuracies, random forest achieves accuracy rates between approximately 93.25% and 94.26% in the yellow category, whereas SVC yields higher accuracy within the range of 95.68%–96.61%. For the non-yellow category, random forest consistently attains commendable accuracy, hovering between roughly 98.23% and 98.57%, whereas SVC shows even greater accuracy, ranging from approximately 98.94%–99.32%, generally surpassing random forest.

The consistency of performance across training sizes and categories is notable in the random forest’s case, where stability is observed. On the other hand, SVC displays slight performance variations, particularly noticeable within the yellow category. In the broader context of comparison, SVC emerges as the more favorable option, showcasing superior performance across most categories and training sizes. While random forest performs admirably in the non-yellow category, it falls short of SVC’s accuracy levels in the yellow category. This analysis underscores the nuanced strengths of each method and the importance of considering the specific problem context when selecting an appropriate machine learning approach.

### 3.3 Discussion of the proposed recognition methods

Up to this point, the task of recognizing yellow-seeded varieties has been effectively accomplished through the application of hyperspectral technology. Now, we would like to delve into the details of the four proposed models.

Beginning with the PLSR-based model, our approach involves identifying yellow-seeded varieties by predicting the RGB values through three essential trilateral parameters obtained from hyperspectral imaging of rapeseed, along with three significant spectral indices. During the process of predicting each R/G/B channel, we extract several noteworthy spectral features that contribute to enhancing the model’s interpretability. It’s important to note that the success of this method relies heavily on the accuracy of RGB calibration. Moving on to the Logit-R model, our strategy revolves around determining yellow-seeded or non-yellow-seeded categorization based on generating probabilities. However, one potential challenge of this model lies in dealing with imbalanced sample data. To address this, when data imbalance is encountered, it’s essential to consider adjusting classification thresholds to ensure accurate results. The optimal hyperspectral feature chosen for both the aforementioned models is determined through a thorough correlation analysis between the R/G/B values and the 23 trilateral parameters and spectral indices derived from a complete band combination. It’s worth mentioning that this approach might potentially omit some information from the original spectral reflectance data. Diverging from the two aforementioned methods, the machine learning models operate differently. In this case, we initially conduct feature dimensionality reduction from a total of 633 features, encompassing all 23 trilateral parameters, 9 spectral indices, and reflectance data from 601 original bands. Subsequently, we select ten key features to input into the random forest and SVC models.

All four models demonstrate high average accuracy rates, showcasing relatively similar performance differences ranging from 93% to 98%. This consistency highlights the feasibility of the framework that combines spectral features with intelligent models for accurately identifying yellow-seeded *B. napus* varieties. Considering factors like ease of operation and comprehensive utilization of information, the SVC model is recommended as an optimal choice for the task of identifying yellow-seeded varieties.

## 4 Discussions and conclusion

Remote sensing technology, recognized as an essential national strategy, finds extensive application across both military and civilian domains. It facilitates the efficient acquisition of spectral data, enabling tasks like land classification and parameter inversion that are challenging for vision-based systems. Hyperspectral imaging technology, a near-Earth remote sensing tool, forms the basis for this advancement. As an innovative method of photoelectric detection and recognition, it integrates spectroscopy with optical imaging, offering a non-destructive and highly efficient alternative to traditional empirical and lab-based approaches for discerning the color of rapeseed seeds. Leveraging the rich spectral and image data inherent to rapeseed samples, this technology holds great promise for agricultural applications.

Comparing yellow seeds with black and brown seeds in Brassica napus reveals that yellow seeds have a thinner seed coat, higher oil content, and better quality. They also have higher protein content in the cake, lower cellulose and polyphenol levels, and higher economic value. Breeding yellow-seed varieties has become a key goal in rapeseed breeding worldwide. However, the complex seed color and inconsistent standards in current identification methods pose challenges. Most researchers use the naked eye or RGB color systems for seed color identification. However, the inconsistent phenotypic color and environmental influences make RGB methods unstable. In contrast, hyperspectral technology, which detects internal seed quality, is less affected by surface color, providing more stable results.

**FIGURE 9:**
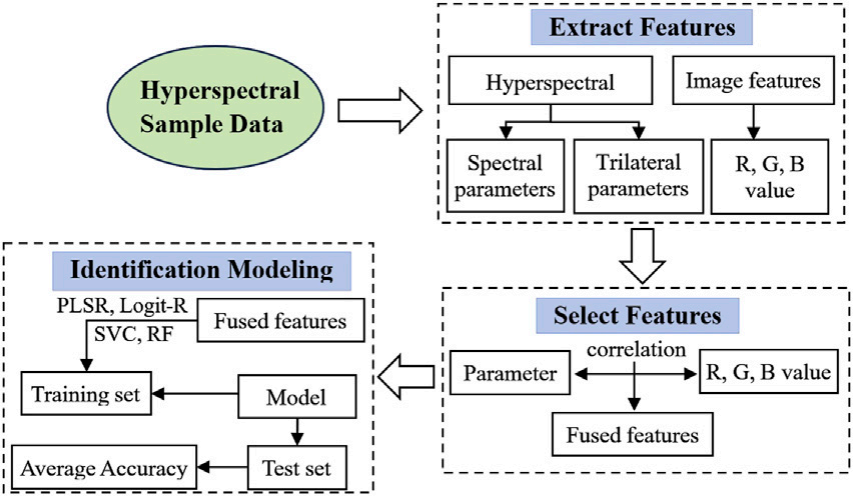
Flowchart of identification modeling.

In this study, we introduce four intelligent models carefully designed to distinguish yellow-seeded rapeseed, as depicted in the model flowchart in Figure 9. The first two models, PLSR and Logit-R, synergize spectral indices with hyperspectral trilateral parameters. This process begins with extracting three spectral indices and 23 trilateral parameters. Through correlation analyses across the R, G, and B color channels of rapeseed seeds, we determine the optimal combinations of these spectral indices and trilateral parameters.

The PLSR model leverages six features derived from three spectral indices and three trilateral parameters, achieving an impressive recognition accuracy (RA) between 92.32% and 96.55% in differentiating yellow from non-yellow seeds. The Logit-R model, which prioritizes the three spectral indices combined with the R channel, achieves a remarkable RA of 98%.

Additionally, we employ two machine learning models-random forest and SVC-to tackle the identification task. Beyond the 23 trilateral parameters and nine optimal spectral indices, we include the original 601 spectral reflectance values in the feature set. Using lasso-penalized logistic regression, we identify ten key features, which serve as input for the random forest and SVC models, achieving an average RA of approximately 98%, with SVC slightly outperforming random forest.

We emphasize that the proposed identification framework for yellow-seeded rapeseed, which integrates classical statistical methods and advanced machine learning tools, demonstrates robust generalizability. This framework is not limited to rapeseed classification but holds significant potential for application to seed classification and identification tasks across a wide range of other crops. By combining hyperspectral feature extraction with predictive modeling techniques, it provides a versatile approach that can adapt to various seed types, accommodating their unique physical and spectral characteristics. This generalizability makes it a valuable tool for advancing precision agriculture and improving the efficiency of crop breeding programs. Additionally, the framework’s ability to extract internal quality information and analyze large-scale data through machine learning models makes it adaptable for various agricultural tasks, including crop variety identification, stress detection, and quality assessment across different agricultural production systems. By customizing the spectral features and models for specific crops, this framework can be effectively extended to other agricultural systems, enhancing precision farming and crop management in diverse contexts.

This study identifies several limitations and proposes future research directions. It suggests integrating machine vision with machine learning for rapeseed color recognition as a cost-effective alternative to hyperspectral feature fusion, which remains expensive. Machine vision, efficient for non-destructive small-target color recognition, contrasts with hyperspectral remote sensing, which excels in large-area identification. A promising approach involves combining hyperspectral remote sensing with intelligent models, establishing a key paradigm for agricultural monitoring. Future work will focus on integrating machine vision and hyperspectral remote sensing to enhance rapeseed color recognition across broader areas.

Second, this study is limited by the small sample size and narrow color range, based on two *B. napus* varieties from a single field trial in Changsha (2020–2021). As an initial exploration of hyperspectral technology and machine learning for yellow-seeded rapeseed identification, it provides valuable insights but requires expansion. Future research will include more rapeseed varieties and account for environmental factors like temperature, humidity, light, and altitude by incorporating multi-year, multi-location data for a comprehensive analysis of seed color variability.

## Data Availability

The raw data supporting the conclusions of this article will be made available by the authors, without undue reservation.
